# To B_12_ or not to B_12_: Five questions on the role of cobalamin in host-microbial interactions

**DOI:** 10.1371/journal.ppat.1007479

**Published:** 2019-01-03

**Authors:** Carol A. Rowley, Melissa M. Kendall

**Affiliations:** Department of Microbiology, Immunology, and Cancer Biology, University of Virginia School of Medicine, Charlottesville, Virginia, United States of America; Tufts University School of Medicine, UNITED STATES

## Introduction

Vitamins are organic compounds that are essential to the health of an organism. Humans cannot synthesize vitamins but obtain vitamins through dietary intake. Cobalamin, or vitamin B_12_, refers to a group of corrinoid molecules that contains a corrin ring with a central cobalt molecule [1]. Cobalamin is a cofactor for the highly conserved enzymes methionine synthase (MetH) and methylmalonyl-CoA mutase (MCM), which function in amino acid synthesis and fatty- and amino acid breakdown, respectively, in both bacteria and mammals [1]; therefore, cobalamin plays a key role in homeostatic functions. Indeed, in humans, cobalamin deficiency can lead to decreased activity of MetH and MCM and result in megaloblastic anemia in addition to severe neurological symptoms. Besides its role as a cofactor for MetH and MCM, cobalamin is also used by many bacteria as a cofactor for additional processes, including metabolism and gene regulation. Cobalamin impacts host–microbe interactions by altering host and bacterial physiology at intestinal and extraintestinal sites. We discuss the current understanding of cobalamin in host–microbiota–pathogen interactions, highlighting recent investigations that deepen our appreciation of this molecule.

### How do mammals acquire cobalamin?

**Fig 1:**
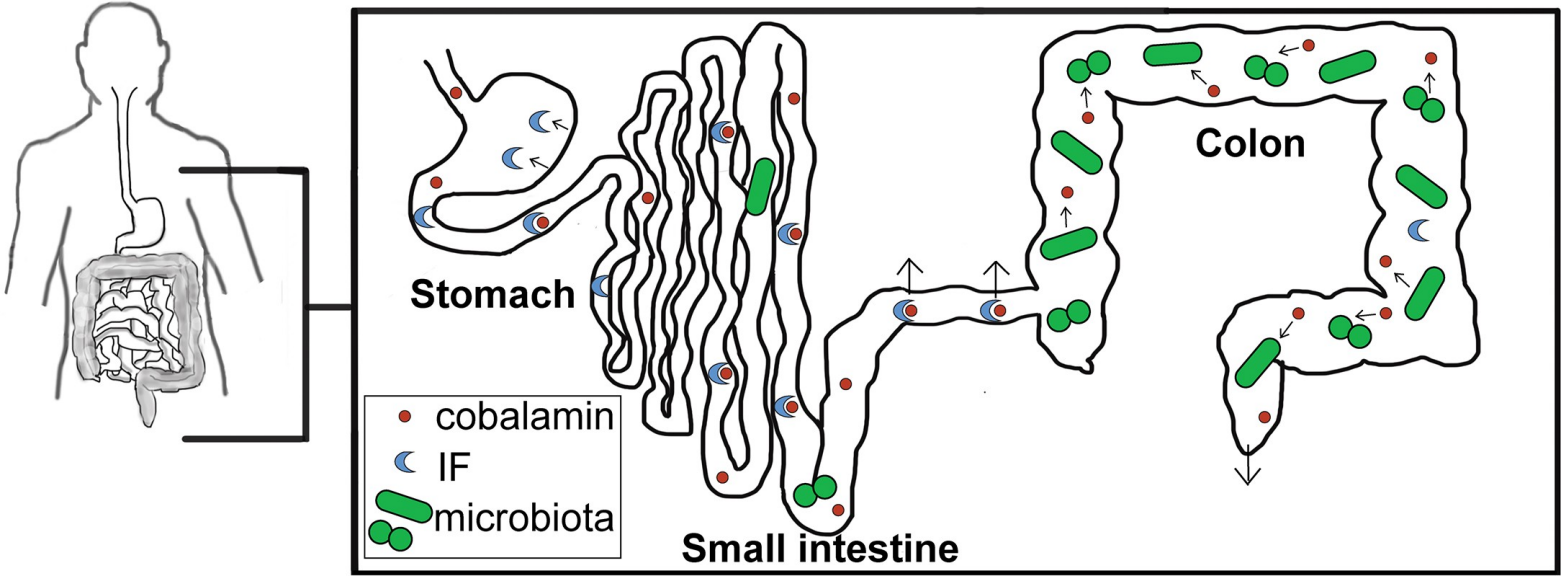
Cobalamin in the healthy human intestinal tract. Ingested food containing cobalamin enters the stomach. IF is produced in the stomach and binds cobalamin in the small intestine. There, cobalamin–IF complexes are absorbed by host enterocytes in the terminal ileum (indicated by outward arrows in small intestine). In the colon, the microbiota take up unabsorbed cobalamin. Additionally, some members of the microbiota produce cobalamin, which can also be taken up by other microbiota members. Cobalamin that remains unabsorbed by the host and not taken up by the microbiota is excreted in stool (outward arrow in colon). IF, intrinsic factor.

Only a limited number of bacteria and archaea generate cobalamin de novo [1]. Moreover, plants and fungi do not require cobalamin; therefore, humans obtain cobalamin through consumption of animal products. During digestion, cobalamin is absorbed in the ileum. Although some colonic bacteria produce cobalamin, humans are not able to uptake cobalamin produced at this location, and thus, the small intestine is the sole site of absorption. The mammalian protein intrinsic factor (IF) is essential to bind and absorb cobalamin. IF is produced in the stomach and binds cobalamin in the small intestine, in which IF-bound cobalamin is subsequently absorbed into circulation [2] (Fig 1).

Mammalian herbivores—including rabbits, mice, rats, and nonhuman primates—practice coprophagy, or eating of feces, to obtain cobalamin because these organisms do not obtain sufficient cobalamin from vegetarian dietary sources. Notably, animal products from ruminants, such as cattle, are excellent dietary sources of cobalamin for humans despite being noncoprophagic herbivores. Ruminants are heavily colonized with cobalamin-producing microbes in the rumen, a specialized organ that allows fermentation of ingested feed, and this cobalamin is later absorbed in the small intestine [3]. Therefore, unlike most mammals, cattle are able to use the cobalamin produced by bacteria that colonize their own gastrointestinal tract without the practice of coprophagy.

### Do humans compete with bacteria for cobalamin?

The ileum harbors a low level of bacteria compared to the colon, and an abnormal expansion of small intestinal bacteria may result in direct host–bacterial competition for cobalamin. Small intestinal bacterial overgrowth (SIBO) is a condition in which the typically low level of 10^3^ bacteria/mL in the small intestine increases to 10^5^ to 10^6^ bacteria/mL, causing chronic diarrhea and malabsorption [4]. Cobalamin deficiency is a common complication of SIBO, potentially resulting from poor nutrient absorption due to diarrhea or by competition for available cobalamin between host IF and resident bacteria. In support of the latter idea, during in vitro growth, members of the genus *Bacteroides* that are common intestinal bacteria outcompete IF for binding to cobalamin [5], suggesting that bacteria interfere with absorption in vivo. Moreover, broad-spectrum tetracycline antibiotic therapy resolves cobalamin deficiency in human subjects [6]. These studies offer proof-of-principle evidence that host–bacterial competition for cobalamin impacts human health.

### What is the interplay among intestinal bacteria for cobalamin?

In the colon, cobalamin availability is determined by two sources—host dietary intake (approximately 50% is not absorbed by the host) [2] and cobalamin generated by select colonic bacteria. Only 25% of bacteria in the gastrointestinal (GI) tract synthesize cobalamin, whereas 80% of bacteria encode cobalamin-dependent enzymes [7]. Therefore, bacteria rely heavily on cobalamin-uptake mechanisms to acquire sufficient levels from the surrounding environment. For example, genome sequencing revealed that 41% of intestinal bacterial strains encode at least three cobalamin-uptake transporters, with some strains encoding up to 17 transporters [7]. To date, over 27 corrinoid transporters have been identified. The importance of encoding multiple transporters is not clear. The transporters were proposed to function redundantly; however, Degnan and colleagues recently reported that *B*. *thetaiotaomicron* expresses three transporters that uptake distinct corrinoids [7]. Even so, not all are required for colonization because a colonization defect was measured only upon deletion of one of these transporters. The decrease in *B*. *thetaiotaomicron* levels in the gut only occurred in the absence of bacteria from the phyla Firmicutes and Actinobacteria [8], which include cobalamin producers, suggesting that cobalamin-generating bacteria support growth of other bacteria in the community. It is reasonable to speculate that interbacterial competition for cobalamin also occurs and influences the composition of the microbiota, though no evidence of this phenomenon has been shown directly.

### Does cobalamin contribute to bacterial pathogenesis?

**Fig 2:**
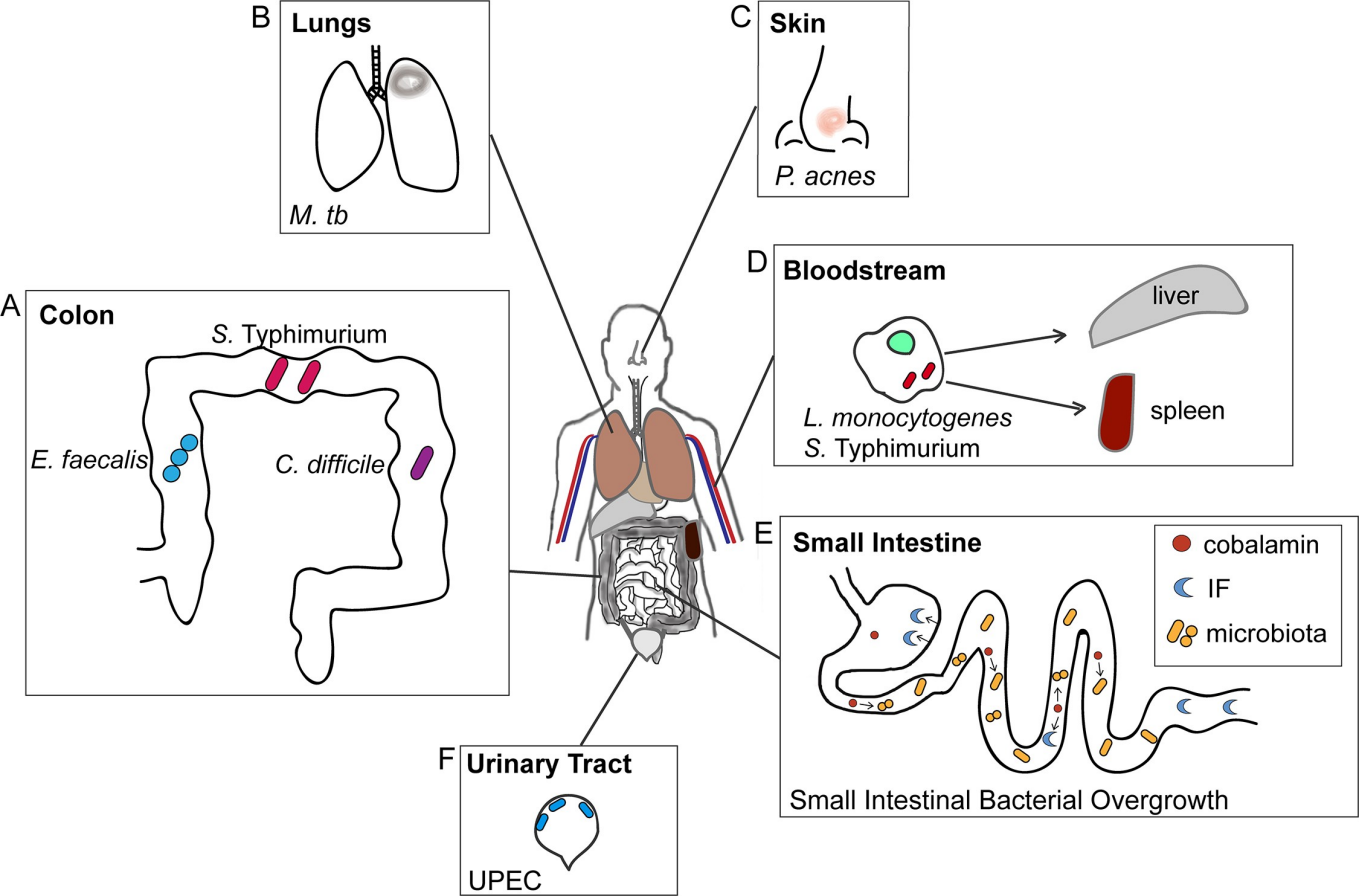
Cobalamin influences pathogenesis at distinct body sites. Each box shows the pathogens demonstrated to rely on cobalamin-dependent processes to grow and/or regulate virulence during infection. (A) *Enterococcus faecalis*, *Salmonella* serovar Typhimurium, and *Clostridium difficile* are shown in the colon. The localization of each strain in the figure is not representative of specific colonic sites of colonization. (B) *Mycobacterium tuberculosis* infection of the lung. (C) *Propionibacterium acnes* colonization of the skin, resulting in an acne pustule on the nose. (D) *Listeria monocytogenes* and *S*. Typhimurium bloodstream infection. Bacteria surviving in macrophages are disseminated to the liver and spleen. (E) An increased number of microbiota members in the small intestine can lead to bacteria cobalamin consumption and competition between IF and bacteria for binding to cobalamin. (F) Uropathogenic *Escherichia coli* colonization of the bladder. IF, intrinsic factor.

Although growth within a host is a requisite for pathogenesis, cobalamin influences the ability of a pathogen to infect a host and cause disease, independent of its role as a cofactor for MetH and MCM. For example, many bacteria encode the *eut* operon that enables growth on ethanolamine (EA), a ubiquitous metabolite in the human body. EA metabolism influences pathogen growth and/or virulence in the intestinal tract (*Salmonella*, *Clostridium*, *Enterococcus*), at sites of extraintestinal dissemination (*Salmonella* and *Listeria*), and in the urinary tract (uropathogenic *Escherichia coli*) [9–17] (Fig 2). In these organisms, the EA ammonia lyase EutBC catalyzes the first step in the breakdown of EA and requires the cobalamin derivative adenosylcobalamin (AdoCbl) for activity. AdoCbl is not a true cofactor because this molecule undergoes irreversible Co-C bond cleavage during EA catabolism, and each round requires a new or readenoslyated AdoCbl molecule for enzyme activity. Moreover, besides encoding metabolic genes, the *eut* operons of the Firmicutes and Enterobacteriaceae also carry distinct regulatory elements that require cobalamin to drive *eut* expression. In the Firmicutes, EA is the signal that initiates phosphorylation of the sensor kinase EutW. This phosphate is then transferred to the noncanonical response regulator EutV (noncanonical because EutV functions post-transcription initiation by binding RNA and preventing formation of transcription-termination structures as opposed to binding DNA). In the absence of cobalamin, the *eut*-encoded small RNA (sRNA) EutX/Rli55 sequesters EutV, thereby inhibiting EutV antitermination activity. The sRNA contains an AdoCbl-binding domain. AdoCbl binding causes a structural change in EutX/Rli55 that results in transcription termination and production of a truncated sRNA that cannot sequester EutV [10, 18]. In the Enterobacteriaceae, *eut* expression requires the transcription factor EutR. Although EutR binds the *eut* promoter in the absence of EA and AdoCbl, both molecules are required for transcription initiation [14, 19]. In enterohemorrhagic *E*. *coli* O157:H7 (EHEC) and *Salmonella*, EutR regulates expression of virulence factors required for host infection and dissemination [11, 13, 14], and, similar to EutR regulation of *eut* expression, transcriptional activation requires EA and AdoCbl. In an analogous manner, cobalamin is required for enzymatic activity of propanediol dehydratase, which breaks down 1,2-propanediol, a metabolite that enhances *Salmonella* growth [20]. Additionally, cobalamin-dependent degradation of 1,2-propanediol releases breakdown product propionate, which promotes *Citrobacter rodentium* virulence during infection of the mammalian gastrointestinal tract [21] (*C*. *rodentium* is a murine pathogen frequently used to model EHEC and enteropathogenic *E*. *coli* infections).

Furthermore, cobalamin has been correlated with influencing gene expression and host survival of *Propionibacterium acnes* and *Mycobacterium tuberculosis*, respectively. For example, in *P*. *acnes*, cobalamin supplementation results in decreased expression of genes encoding cobalamin synthesis and increased production of porphyrins [22], which has been linked to inflammation in acne. Additionally, the obligate pathogen *M*. *tb* infects the human lung. Cobalamin transport and synthesis impact host survival and *M*. *tb* growth, respectively, although none of the three cobalamin-dependent *M*. *tb* enzymes are required for growth in vivo or in vitro [23]. Further study is required to elucidate the specific pathways involved in cobalamin-mediated pathogenesis during *P*. *acnes* and *M*. *tb* infection.

### Does cobalamin play a role in host defense against infection?

It is clear that cobalamin impacts the ability of a pathogen to establish infection and influence disease progression; however, the contribution of cobalamin to the host’s ability to defend against infection is less understood. Recently, Mottram and colleagues reported that IF mediates a protective effect against *Salmonella* and *C*. *rodentium* infection. In this study, mice with a genetic deletion in IF production displayed worsened pathology compared to wild-type mice and increased mortality during *Salmonella* and *C*. *rodentium* infection [24]. These data suggest that cobalamin absorption in a host promotes proper defense against pathogenic challenge. To better understand why IF-deplete mice exhibit profound susceptibility to enteric infection, Mottram and colleagues performed analysis of immune cell populations in IF-deplete mice prior to infection. These studies demonstrated slightly elevated percentages of CD4+ and CD8+ T cells in IF-deplete mice compared to wild-type mice, suggesting distinct immune responses in these animals without clarifying what caused the mortality differences. Further studies are necessary to identify potential immune changes during enteric infection in mice lacking cobalamin and to improve understanding of the role of cobalamin in immune function.

## Conclusion

Recent studies have built on our understanding of the critical role of cobalamin as an enzymatic cofactor to include newly identified roles in gene expression, pathogenesis, and immune function. Elucidating the vast influence of cobalamin in host–microbiota–pathogen interactions will expand our understanding of host–microbial homeostasis and lead to creative new opportunities to disrupt pathogenesis.

## References

[1] Roth J, Lawrence J, Bobik T. COBALAMIN (COENZYME B12): Synthesis and Biological Significance. Annual Review of Microbiology. 1996;50:137–81. 10.1146/annurev.micro.50.1.137 . 8905078

[2] Stabler SP, Allen RH. Vitamin B12 deficiency as a worldwide problem. Annual Review of Nutrition. 2004;24:299–326. 10.1146/annurev.nutr.24.012003.132440 . 15189123

[3] Girard CL, Santschi DE, Stabler SP, Allen RH. Apparent ruminal synthesis and intestinal disappearance of vitamin B12 and its analogs in dairy cows. Journal of Dairy Science. 2009;92:4524–9. 10.3168/jds.2009-2049 19700714

[4] Dukowicz AC, Lacy BE, Levine GM. Small intestinal bacterial overgrowth: a comprehensive review. Gastroenterology & hepatology. 2007;3:112–22. . 21960820 PMC3099351

[5] Wexler AG, Schofield WB, Degnan PH, Folta-Stogniew E, Barry NA, Goodman AL. Human gut Bacteroides capture vitamin B12 via cell surface-exposed lipoproteins. eLife. 2018;7 10.7554/eLife.37138 30226189 PMC6143338

[6] Suter PM, Golner BB, Goldin BR, Morrow FD, Russell RM. Reversal of protein-bound vitamin B12 malabsorption with antibiotics in atrophic gastritis. Gastroenterology. 1991;101:1039–45. . 1889697 10.1016/0016-5085(91)90731-y

[7] Degnan PH, Barry NA, Mok KC, Taga ME, Goodman AL. Human gut microbes use multiple transporters to distinguish vitamin B₁₂ analogs and compete in the gut. Cell host & microbe. 2014;15:47–57. 10.1016/j.chom.2013.12.007 . 24439897 PMC3923405

[8] Goodman AL, McNulty NP, Zhao Y, Leip D, Mitra RD, Lozupone CA, et al Identifying Genetic Determinants Needed to Establish a Human Gut Symbiont in Its Habitat. Cell Host & Microbe. 2009;6:279–89. 10.1016/j.chom.2009.08.003 . 19748469 PMC2895552

[9] Maadani A, Fox KA, Mylonakis E, Garsin DA. Enterococcus faecalis mutations affecting virulence in the Caenorhabditis elegans model host. Infection and immunity. 2007;75:2634–7. 10.1128/IAI.01372-06 . 17307944 PMC1865755

[10] Mellin JR, Koutero M, Dar D, Nahori M-A, Sorek R, Cossart P. Sequestration of a two-component response regulator by a riboswitch-regulated noncoding RNA. Science. 2014;345:940–3. 10.1126/science.1255083 . 25146292

[11] Anderson CJ, Clark DE, Adli M, Kendall MM. Ethanolamine Signaling Promotes Salmonella Niche Recognition and Adaptation during Infection. PLoS Pathog. 2015;11 10.1371/journal.ppat.1005278 . 26565973 PMC4643982

[12] Thiennimitr P, Winter SE, Winter MG, Xavier MN, Tolstikov V, Huseby DL, et al Intestinal inflammation allows Salmonella to use ethanolamine to compete with the microbiota. Proceedings of the National Academy of Sciences. 2011;108:17480–5. 10.1073/pnas.1107857108 . 21969563 PMC3198331

[13] Kendall MM, Gruber CC, Parker CT, Sperandio V. Ethanolamine controls expression of genes encoding components involved in interkingdom signaling and virulence in enterohemorrhagic escherichia coli O157:H7. mBio. 2012;3 10.1128/mBio.00050-12 . 22589288 PMC3372972

[14] Luzader DH, Clark DE, Gonyar LA, Kendall MM. EutR is a direct regulator of genes that contribute to metabolism and virulence in enterohemorrhagic escherichia coli o157: H7. Journal of Bacteriology. 2013;195:4947–53. 10.1128/JB.00937-13 . 23995630 PMC3807496

[15] Sintsova A, Smith S, Subashchandrabose S, Mobley HL. Role of Ethanolamine Utilization Genes in Host Colonization during Urinary Tract Infection. Infection and immunity. 2018;86:e00542–17. 10.1128/IAI.00542-17 . 29229730 PMC5820945

[16] Nawrocki KL, Wetzel D, Jones JB, Woods EC, McBride SM. Ethanolamine is a valuable nutrient source that impacts *Clostridium difficile* pathogenesis. Environmental Microbiology. 2018 10.1111/1462-2920.14048 29349925 PMC5903940

[17] Gonyar LA, Kendall MM. Ethanolamine and choline promote expression of putative and characterized fimbriae in enterohemorrhagic Escherichia coli O157:H7. Infect Immun. 2014;82(1):193–201. 10.1128/IAI.00980-13 24126525 PMC3911853

[18] DebRoy S, Gebbie M, Ramesh A, Goodson JR, Cruz MR, van Hoof A, et al Riboswitches. A riboswitch-containing sRNA controls gene expression by sequestration of a response regulator. Science. 2014;345(6199):937–40. 10.1126/science.1255091 25146291 PMC4356242

[19] Roof DM, Roth JR. Autogenous regulation of ethanolamine utilization by a transcriptional activator of the eut operon in Salmonella typhimurium. J Bacteriol. 1992;174(20):6634–43. 1328159 10.1128/jb.174.20.6634-6643.1992 PMC207641

[20] Faber F, Thiennimitr P, Spiga L, Byndloss MX, Litvak Y, Lawhon S, et al Respiration of Microbiota-Derived 1,2-propanediol Drives Salmonella Expansion during Colitis. PLoS Pathog. 2017;13:e1006129 10.1371/journal.ppat.1006129 28056091 PMC5215881

[21] Connolly JPR, Slater SL, O'boyle N, Goldstone RJ, Crepin VF, Gallego DR, et al Host-associated niche metabolism controls enteric infection through fine-tuning the regulation of type 3 secretion. 10.1038/s41467-018-06701-4 30305622 PMC6180029

[22] Kang D, Shi B, Erfe MC, Craft N, Li H. Vitamin B 12 modulates the transcriptome of the skin microbiota in acne pathogenesis. Sci Transl Med. 2015. 10.1126/scitranslmed.aab2009 PMC6049814 26109103

[23] Gopinath K, Moosa A, Mizrahi V, Warner DF. Vitamin B12 metabolism in Mycobacterium tuberculosis. Future Microbiology. 2013;8:1405–18. 10.2217/fmb.13.113 24199800

[24] Mottram L, Speak AO, Selek RM, Cambridge EL, McIntyre Z, Kane L, et al Infection Susceptibility in Gastric Intrinsic Factor (Vitamin B12)-Defective Mice Is Subject to Maternal Influences. mBio. 2016;7:e00830–16. 10.1128/mBio.00830-16 . 27329747 PMC4916386

